# Rheological Properties of Wheat Flour Modified by Plasma-Activated Water and Heat Moisture Treatment and *in vitro* Digestibility of Steamed Bread

**DOI:** 10.3389/fnut.2022.850227

**Published:** 2022-03-17

**Authors:** Miaomiao Shi, Yanqiu Cheng, Fei Wang, Xiaolong Ji, Yanqi Liu, Yizhe Yan

**Affiliations:** ^1^Henan Key Laboratory of Cold Chain Food Quality and Safety Control, College of Food and Bioengineering, Zhengzhou University of Light Industry, Zhengzhou, China; ^2^Lanzhou Quality Supervision Center Limited, China Grain Reserves Group Ltd. Company, Lanzhou, China

**Keywords:** wheat flour, heat-moisture treatment, plasma-activated water, steamed bread, *in vitro* digestibility

## Abstract

The study investigated the effects of plasma-activated water (PAW) and heat moisture treatment (HMT) on the rheological properties of wheat flour and the *in vitro* digestibility of steamed bread partially replaced by the modified wheat flour. After HMT, the gelatinization temperature of wheat flour increased and the gelatinization enthalpy reduced. The solubility and swelling power of wheat flour increased after the heat-moisture treatment. The solubility of modified flour after PAW-HMT treatment was lower than that of distilled water (DW)-HMT at the same temperature. The wheat flour with HMT had higher storage modulus (G') and loss modulus (G“), and had better ductility and deformability. Common wheat flour was partially replaced by modified flour to make steamed bread. The results indicated that the volume, height, diameter and specific volume of steamed bread were significantly decreased with the addition of HMT flour. However, the hardness, viscosity and chewiness increased significantly. The resistant starch content of steamed bread with the modified wheat flour increased. The results provide new insights for the development of new functional steamed bread.

## Introduction

Wheat is the largest food crop globally, accounting for one-third of the world's total annual production. It is the main food material in China and many countries in the world. Approximately 35% of the world's population eat wheat-based food, such as bread, biscuits, noodles and steamed bread (1). Wheat flour is made up of starch, lipids, proteins and non-starch polysaccharides. It is an important main material for noodles, steamed bread, biscuits and other flour products.

In order to improve the functional characteristics of wheat flour and broad its application in food, it is necessary to modify wheat flour. Heat moisture treatment (HMT) can be used to improve the undesirable properties of flour or starch. It can promote the change of starch chain structure arrangement in amorphous and crystalline regions of granules, resulting in granule expansion and changes in starch crystallinity, pasting characteristics, thermal stability and other properties (2). Chen et al. (3) studied the physical and chemical properties of wheat flour and wheat starch after HMT, and found that the gelatinization temperature of wheat flour and wheat starch increased after HMT, resulting in the formation of starch-lipid complex. The double helix structure was dissociated, which promoted the interaction between polymer chains. Compared with wheat starch, wheat flour treated by HMT showed higher resistance and slowly digestible starch content. Therefore, heat-moisture treatment is an effective method to improve the functional properties of wheat starch and flour.

Plasma technology is a green and non-thermal treatment technology, which has the advantages of safety, short time, no residue and simple equipment. The water treated by plasma technology is called plasma-activated water (PAW), which is a liquid obtained by plasma discharge in water or on the surface of water. It has the characteristics of low pH, high conductivity and high REDOX potential (4). The main research objects of cold plasma in the modification of food components are starch and protein. The active chemical substances generated by gas ionization can change the structure of these macromolecules, promote the cross-linking and depolymerization between molecules and the formation of new functional groups. So as to achieve the effect of changing the functional properties of food (5, 6). In recent years, researchers have used plasma technology to modify starch or flour in order to obtain good quality products. Yan et al. (7) found that the physical and chemical properties of corn starch were significantly changed with PAW treatment, and the content of resistant starch increased, and the *in vitro* digestibility decreased. Misra et al. (8) found that the protein molecular hydrogen bond strength of low-gluten wheat flour was enhanced, and the protein secondary structure was more stable after treatment with atmospheric pressure cold plasma. Chaple et al. (9) found that the crystallinity and enthalpy change of wheat flour decreased and the viscosity increased after treatment with atmospheric cold plasma. This may be caused by PAW-induced depolymerization of starch in wheat flour. They believed that the plasma functionalized flour or starch could be used as an ingredient in product formulations of various foods. However, the related studies of PAW mainly focused on the experimental methods, and there were few studies on the mechanism of reaction. In this experiment, the effect of PAW combined with HMT on the properties of wheat flour and the quality of steamed bread after partial replacement of common wheat flour. This study can expand the application scope of plasma technology, the preparation methods of high-quality wheat flour, and provide reference for the development of new functional flour products.

## Materials and Methods

### Materials

Wheat flour (WF) was obtained from COFCO Grain and Oil Co., Ltd. (Zhengzhou, China). Porcine pancreatin (P7545, 8 × USP) and amyloglucosidase (A7095, 300 U/mL) were purchased from Sigma in the United States. Glucose oxidase peroxidase (GOPOD) glucose kit was purchased from Megazyme Co., Ltd (Ireland). Absolute ethanol was provided by Fuyu Chemical Co., Ltd. (Tianjin, China). The chemical reagents used were all analytical grade.

### Preparation of Plasma-Activated Water

Atmospheric pressure plasma jet (APPJ) device was used (Easton Geake Automation Equipment Co., Ltd., Shenzhen, China) to prepare plasma-activated water (PAW). PAW can be obtained by treating every 100 mL of distilled water with plasma jetting for 2 min. The plasma jet probe was located directly above the water surface at a distance of 56.25 mm. The high frequency of the power supply was 40 kHz, and the high voltage was 5 kV.

### HMT of WF With PAW

Wheat flour (40 g, dry basis) was placed in an air oven (40°C) and dried until the moisture content was about 5%. Then, the moisture content of wheat flour was adjusted to 15 and 25% with plasma-activated water. Distilled water (DW) was regarded as a reference. The flour samples were put into the hydrothermal reactor (Yikai Instrument Equipment Co., Ltd., Shanghai, China) to heat at 100°C for 1 h in an air oven after equilibrating at 25°C for 12 h. The treated flour sample was dried at 40°C for 12 h in an air oven, then ground to pass through 100-mesh sieve for further analysis. The samples were denoted as DW-15%, DW-25%, PAW-15% and PAW-25% on the basis of the moisture content of DW and PAW.

### Differential Scanning Calorimetry

The flour sample (30%, w/w) was accurately weighed into an aluminum pan, and equilibrated at room temperature for 12 h. An empty aluminum pan was used as a control. Heating of each sample was carried out from 20 to 140°C at 10°C/min rate. The onset (To), peak (Tp), conclusion (Tc) transition temperatures and enthalpy changes (ΔH) were determined using the DSC differential scanning calorimeter (DSC Q20, TA instrument Inc., USA).

### Solubility and Swelling Power

Solubility and swelling power were determined and calculated by the method suggested by Colussi et al. (10) with a slight modification. The flour samples (0.5 g, dry basis) and 25 mL of distilled water were heated at 90°C for 30 min. Then the cooled solution was centrifuged (352 g) for 20 min. The supernatant was dried at 105°C to obtain the weight of the dried solid. At the same time, the wet precipitate in the centrifuge tube was weighed.

### Rheological Properties of Wheat Flour

The rheological behavior of wheat flour was measured by the Discovery rheometer (Discovery HR-1, TA instrument Inc., USA). The flat panel system was used to test, with a probe diameter (40 mm) and a gap (1 mm). Before measurement, the sample was equilibrated at 25°C for 5 min. The sample preparation referred to the method of Solaesa et al. (11), with appropriate modifications. The prepared gel was placed on the plate mold, and the edge was trimmed.

The oscillation frequency mode was adopted. The temperature was constant at 25°C, and the scanning frequency was set at 1 Hz. The shear strain was from 0.01 to 100%. The relation curves of the storage modulus (G') and loss modulus (G”) with the strain amplitude were measured.

The dynamic rheological data of the sample were measured in the frequency sweep range of 0.1–20.0 Hz with the strain of 1% within the linear viscoelastic region at 25°C, and the storage modulus (G'), loss modulus (G“) and loss tangent (tan δ) of the flour gel were measured.

### Preparation of the Steamed Bread

The wheat flour (100 g) was mixed with a well-mixed yeast solution (1 g dry yeast and a certain amount of water, 80% of the Farinograph water absorption). The mixture was put into the dough mixer (EGK205 L, Rongshida Small Home Appliance Co., Ltd., Hefei, China) for a certain period of time (the mixing time was determined by the stability time of the dough) until the dough was smooth. Then the dough was pressed 15 times in a noodle machine to squeeze out the bubbles. Afterwards, the dough was put in the fermenting box (HWS-080, Jinghong Experimental Equipment Co. Ltd., Shanghai, China) for 1 h at 35°C. Steamed breads (50 g of each dough) were steamed in a steamer for 30 min, and then cooled at 25°C for 1 h. Steamed bread was made with 20% or 30% (w/w) DW-15% and PAW-15% samples instead of wheat flour, respectively. The samples were denoted as B-DW-20%, B-DW-30%, B-PAW-20% and B-PAW-30% on the basis of the addition amount of HMT flour.

### Determination of Basic Indicators

The weight and diameter of steamed bread were measured according to GB/T 5991-2018 (Chinese National Standard). The volume of steamed breads was measured according to the method of Shi et al. (12). Specific volume and spread ratio measurements followed the methods of Liu et al. (13) with slight modifications. The ratio of the volume to weight was the specific volume of the steamed bread, and the spread ratio was the ratio of the diameter to the height of the steamed bread.

### Texture Profile Analysis

The texture analysis of the steamed bread was carried out with the TA-XT plus texture analyzer (Stable Micro Systems Ltd., Godalming, UK). According to the method of Gao et al. (14), the central part of the steamed bread was cut into 20 mm slices. The pretest, testing and posttest speeds were 3.00, 1.00, and 1.00 mm/s, respectively. The strain was 50% and the trigger force was 5 g. The hardness, springiness, cohesion, viscosity and chewiness of the steamed bread were measured.

### *In vitro* Digestibility Test

Steamed bread was cut into small pieces and dried in a vacuum freeze dryer for 12 h. The freeze-dried steamed bread was crushed through a 100-mesh sieve. The *in vitro* digestibility of the steamed bread was carried out according to the method of Englyst et al. (15), as modified by Shi et al. (12). The steamed bread flour (200 mg, dry basis) was accurately weighed and mixed with sodium acetate buffer solution (4 mL, 0.1 mol/L). Then, the porcine pancreatin and amyloglucosidase mixture (1 mL) was added to the sample and reacted in a water bath oscillator (37°C, 22 g). Ethanol (4 mL, 70%) was mixed with the above reaction solution (0.1 mL) to inactivate at 20 and 120 min, respectively, and centrifuged for 10 min (352 g). Then, supernatant (1 mL) was added with GOPOD reagent (3 mL) in water bath to develop color (45°C, 20 min). Its absorbance was measured at 510 nm. In addition, standard glucose solution (0.1 mL) was taken as the standard glucose and distilled water (0.1 mL) as the blank product. According to the absorbance of sample and standard glucose, the contents of rapidly digestible starch (RDS), slowly digestible starch (SDS) and resistant starch (RS) were calculated, respectively.

### Statistical Analysis

All experiments were repeated three times, and the data were expressed as mean values ± standard deviations. Statistical analysis used SPSS 26.0 software, and analysis of variance used Duncan's multiple comparison method for significance test (*P* < 0.05). The graphs were all obtained from Origin 9.0 software.

## Results and Discussion

### Thermal Properties

**Figure 1:**
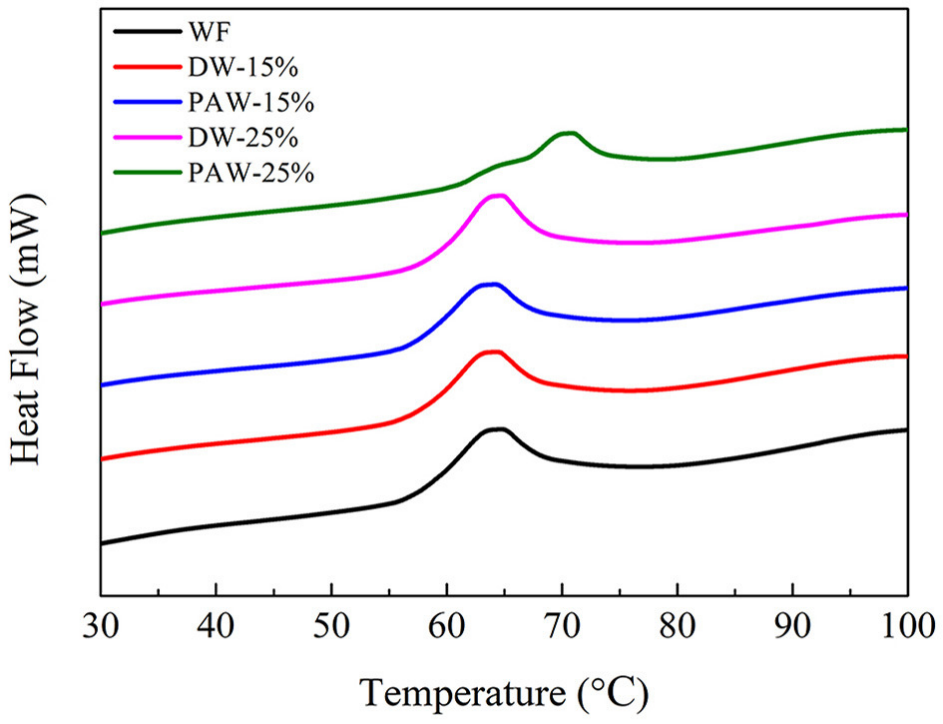
Thermal properties of wheat flour.

The gelatinization parameters of wheat flour with heat-moisture treatment are shown in Figure 1 and Table 1. After wheat flour treated with HMT, the gelatinization temperature (To, Tp, and Tc) of wheat flour increased. It may be due to the redirection of starch granules and the recombination of the crystalline structure. On the other hand, it was due to the enhanced interaction between amylose and amylopectin. Therefore, a higher temperature was required to destroy the crystal. The gelatinization enthalpy indicates the reduction of starch double helix order during the gelatinization process. Compared with natural wheat flour, the ΔH of flour with HMT treatment was lower. The decrease in ΔH during the HMT treatment indicates that some double helixes crystallization of the particles may be decomposed under HMT. The ΔH of PAW-25% sample was the lowest. It may be due to the formation of more heterogeneous crystallites which caused by the acid degradation from plasma activated water (16, 17).

**Table 1:** Thermal properties of wheat flour.

**Sample**	**To (**°**C)**	**Tp (**°**C)**	**Tc (**°**C)**	**ΔH (J/g)**
WF	57.72 ± 0.11^a^	63.82 ± 0.10^a^	78.00 ± 1.22^a^	7.38 ± 0.32^b^
DW-15%	58.34 ± 1.03^ab^	64.04 ± 0.98^a^	79.24 ± 6.02^a^	6.75 ± 0.51^b^
PAW-15%	57.82 ± 0.48^ab^	64.02 ± 0.65^a^	78.26 ± 1.43^a^	7.06 ± 0.17^b^
DW-25%	58.86 ± 0.39^b^	64.28 ± 0.17^a^	80.64 ± 0.19^a^	7.17 ± 0.11^b^
PAW-25%	64.92 ± 0.46^c^	70.24 ± 0.18^b^	79.41 ± 2.62^a^	5.21 ± 1.12^a^

*Values with different lowercase letters in the same line indicate a significant difference at p < 0.05*.

### Solubility and Swelling Power

**Figure 2:**
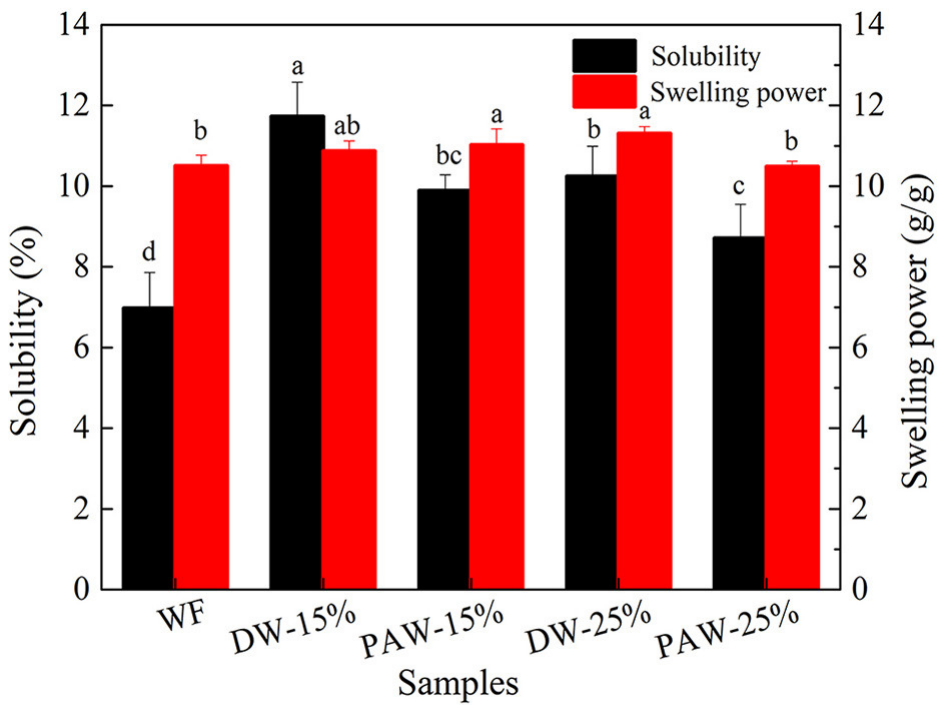
Solubility and swelling power of wheat flour. Different lowercase letters indicate significant differences at *p* < 0.05.

The solubility and swelling power of wheat flour are shown in Figure 2. The solubility and swelling power of modified flour were higher than those of native flour. It may be due to the destruction of the granule molecular structure when starch was gelatinized. The interaction between starch and water was enhanced, which increased the swelling of wheat flour (18). Deka and Sit (19) found that the structural reorganization of taro starch during microwave and HMT may promote the formation of long-chain amylose molecules, thus the swelling power was increased. The increased solubility may be due to the partial disruption of the amylopectin helices and increased leaching capacity of amylose from damaged granules. Under the same conditions, the solubility of PAW-HMT flour was lower than that of DW-HMT flour. This may be due to the increased amylose content and enhanced interaction between starch and protein with the action of PAW. In addition, after protein denaturation, the solubility of the sample will also decrease.

### Rheological Properties

**Figure 3:**
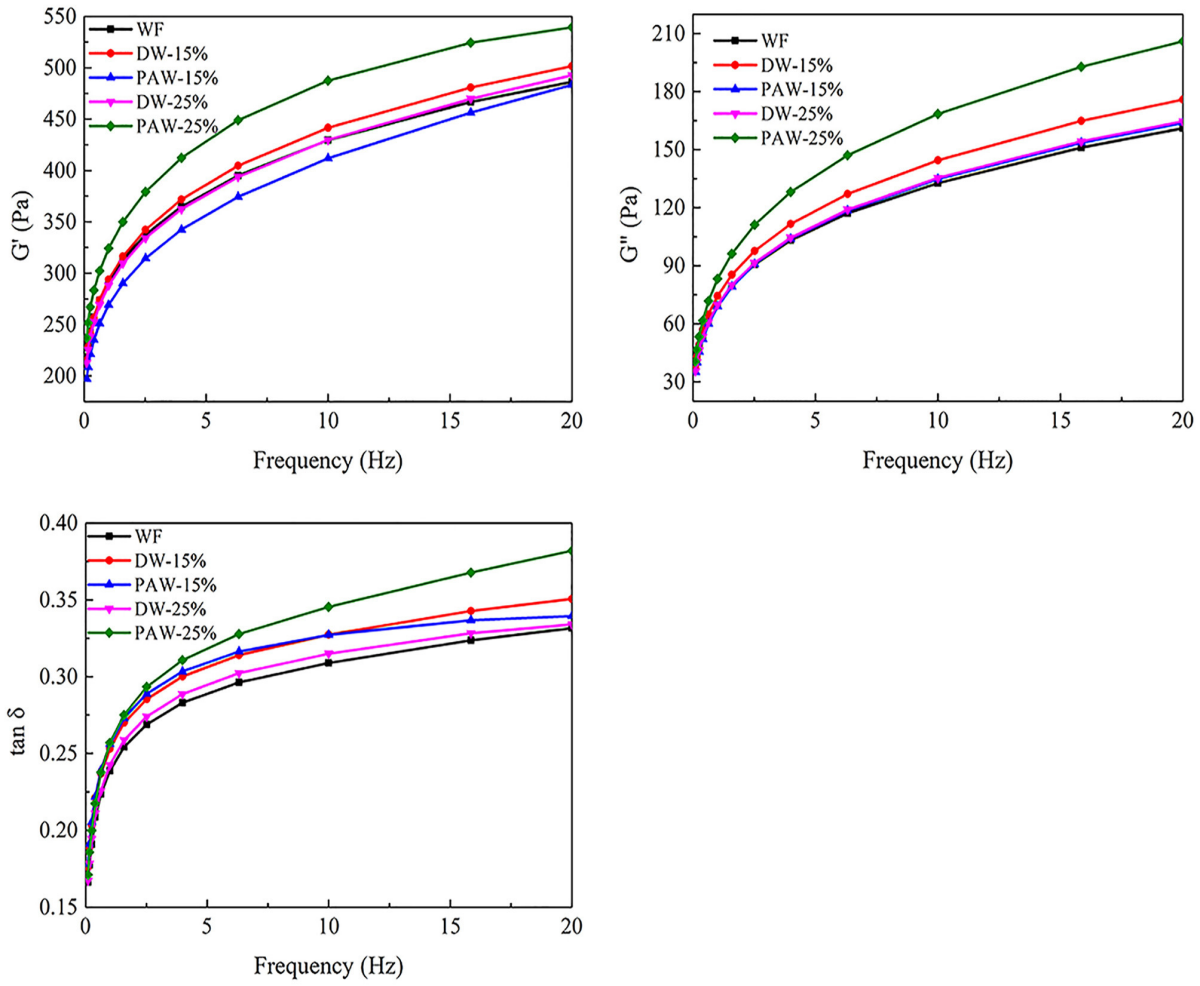
Rheological properties of wheat flour. Storage modulus (G'), loss modulus (G”), dynamic loss tangent (tan δ).

The rheological properties of wheat flour with heat-moisture treatment were measured by dynamic oscillation test. The storage modulus (G') is used to represent the elastic characteristics of the gel and reflects the degree of deformation of the sample. The loss modulus (G”) represents the viscosity properties of the gel and reflects the resistance of the sample to flow. As shown in Figure 3, the storage modulus (G′) and loss modulus (G″) of wheat flour were directly proportional to frequency. The value of G′ was higher than that of G″, indicating that the elasticity of flour was greater than the viscosity. The viscoelasticity of wheat flour increased after heat-moisture treatment. It may be due to structural changes in the main components of wheat flour with HMT, including starch gelatinization and aging after cooling, as well as protein denaturation during heating, resulting in improved viscoelasticity of flour (13). Yang et al. (20) proposed that starch molecular chains were degraded due to heat treatment. It was conducive to the rearrangement of starch molecules, forming a continuous gel network structure, thereby enhancing its gel strength. The G' and G“ of wheat flour treated with DW-15% were higher than those of PAW-15%. The higher G′ means the higher the strength and hardness of the gel. The decrease of G′ of wheat flour treated with PAW-HMT may be due to the weakening of the gel strength of wheat flour due to the acid degradation effect of PAW (7). However, the G' and G” of wheat flour treated with PAW-25% were higher than those of the DW-25%. It may be due to the enhanced interaction between starch and protein under the action of PAW and a higher water content.

Tan δ is the ratio of viscosity modulus to elastic modulus (G“/G'). The higher the value of tan δ represented the stronger the viscosity and fluidity. The smaller the tan δ value represented the stronger the elasticity and solid properties. It can be seen from Figure 2 that the tan δ values of all samples were much <1. It showed that the wheat flour paste was more elastic and behaved more like a solid (21). The tan δ value of the treated wheat flour paste increased, indicating that the treated wheat flour paste had a softer and less solid structure (22). These results showed that each treatment can delay the tendency of internal molecular binding of wheat flour paste, thereby improving the stability of the paste (23).

### Analysis of Basic Indicators of Steamed Bread

The effects of the modified flours on the basic indicators of steamed bread are shown in Table 2. In comparison with the control, the quality of steamed bread with modified flour changed. The volume, height and diameter of steamed bread decreased significantly after the addition of modified flour. It indicated that the modified flour could hinder the fermentation of dough, and the steamed bread could not form a better gluten network structure. The protein was denatured under the heat condition, and the hydrogen bonds that stabilize its structure were destroyed. Some hydrophobic residues and reaction groups were leaked out due to the extension of molecules, leading to the aggregation reaction between the protein molecules. The gluten structure had no able to support bubble expansion in dough, so the gas holding capacity of steamed bread was inhibited, and the expansion degree of steamed bread was limited. Li et al. (24) found that the tensile strength and tensile distance of noodles were lower than those of natural noodles after adding a certain proportion of heat-moisture treatment flour. The noodles were easy to break, which was also related to protein denaturation. The mass, volume, height, diameter and specific volume of steamed bread with PAW-HMT flour were lower than that of DW-HMT flour under the same conditions. It may be due to the acid hydrolysis of starch and protein molecules in flour by acidic substances in PAW. The performance of B-PAW-30% was more obvious, which was related to the increase of acidic substances in PAW.

**Table 2:** Basic indicators of steamed bread.

**Samples**	**Weight (g)**	**Volume (cm^**3**^)**	**Height (cm)**	**Diameter (cm)**	**Specific volume (mL/g)**	**Spread ratio**
Control	51.20 ± 0.08^a^	140.00 ± 0.00^a^	4.10 ± 0.14^a^	7.10 ± 0.14^a^	2.73 ± 0.00^a^	1.73 ± 0.09^b^
B-DW-20%	50.60 ± 0.16^b^	107.50 ± 3.54^b^	3.75 ± 0.07^b^	6.70 ± 0.00^b^	2.12 ± 0.06^b^	1.79 ± 0.03^a^
B-PAW-20%	50.16 ± 0.30^c^	99.50 ± 0.71^c^	3.40 ± 0.14^c^	6.50 ± 0.00^c^	1.98 ± 0.00^c^	1.91 ± 0.08^a^
B-DW-30%	50.48 ± 0.08^bc^	100.50 ± 0.00^c^	3.75 ± 0.07^b^	6.65 ± 0.07^bc^	1.99 ± 0.00^c^	1.77 ± 0.05^a^
B-PAW-30%	49.70 ± 0.01^d^	98.00 ± 1.41^c^	3.40 ± 0.14^c^	6.30 ± 0.00^d^	1.97 ± 0.03^c^	1.85 ± 0.08^a^

*Values with different lowercase letters in the same line indicate a significant difference at p < 0.05*.

### Texture of Steamed Bread

The effect of modified flour on the steamed bread texture is shown in Table 3. With the addition of modified flour, the hardness, viscosity and chewiness of steamed bread increased significantly, while the elasticity and cohesion decreased. The texture properties of steamed bread with PAW-HMT modified flour changed obviously. Especially, when 30% PAW-15% flour was added, the hardness, viscosity and chewiness of steamed bread reached the maximum. According to the results of the basic indicators of steamed bread, it can be seen that the addition of modified flour restricted the formation of network structure in dough, and weakened the expansion degree of steamed bread. These results led to an increase in the hardness of steamed bread. The results of HMT of highland barley starch showed that the HMT induced amylopectin molecular side chains to break and form short starch chains, which reduced the molecular weight of starch and increased the amylose content in the starch (25). After the flour was depolymerized by the acidic substance in PAW, the content of amylose in the flour increased after the heat treatment. Some research reports that the higher the amylose content, the faster the retrogradation rate and speed of starch could result in less than ideal flour products (26). As the most abundant starch, it also affects the properties of steamed bread. Amylopectin was destroyed to amylose after HMT, and the multiple gelatinization of starch will aggravate the hardening of steamed bread. In addition, the protein was denatured and the gluten protein was destroyed in the wheat flour with PAW-HMT. The formation of spatial network structure of steamed bread with PAW-15% addition was weak, resulting in a decrease in the volume of steamed bread and an increase in hardness. So, the hardness and chewability of steamed bread increased significantly.

**Table 3:** Texture determination of steamed bread.

**Samples**	**Hardness (g)**	**Springiness (g. s)**	**Cohesiveness**	**Adhesiveness**	**Chewiness (g)**
Control	8,403.29 ± 531^d^	0.89 ± 0.02^a^	0.62 ± 0.00^a^	5,213.86 ± 322.35^a^	4,597.05 ± 189.77^d^
B-DW-20%	10,086.52 ± 277.21^c^	0.90 ± 0.04^a^	0.61 ± 0.00^ab^	6,122.59 ± 180.50^c^	5,485.44 ± 57.95^c^
B-PAW-20%	12,779.68 ± 56.02^b^	0.84 ± 0.03^a^	0.61 ± 0.00^ab^	7,785.68 ± 26.45^b^	6,913.83 ± 20.32^b^
B-DW-30%	10,785.01 ± 368.30^c^	0.87 ± 0.05^a^	0.60 ± 0.01^a^	6,496.42 ± 65.40^c^	5,611.66 ± 249.98^c^
B-PAW-30%	15,560.63 ± 68.98^a^	0.81 ± 0.01^a^	0.60 ± 0.01^a^	9,317.38 ± 79.82^a^	7,496.76 ± 338.44^a^

*Values with different lowercase letters in the same line indicate a significant difference at p < 0.05*.

### *In vitro* Digestibility Test

**Figure 4:**
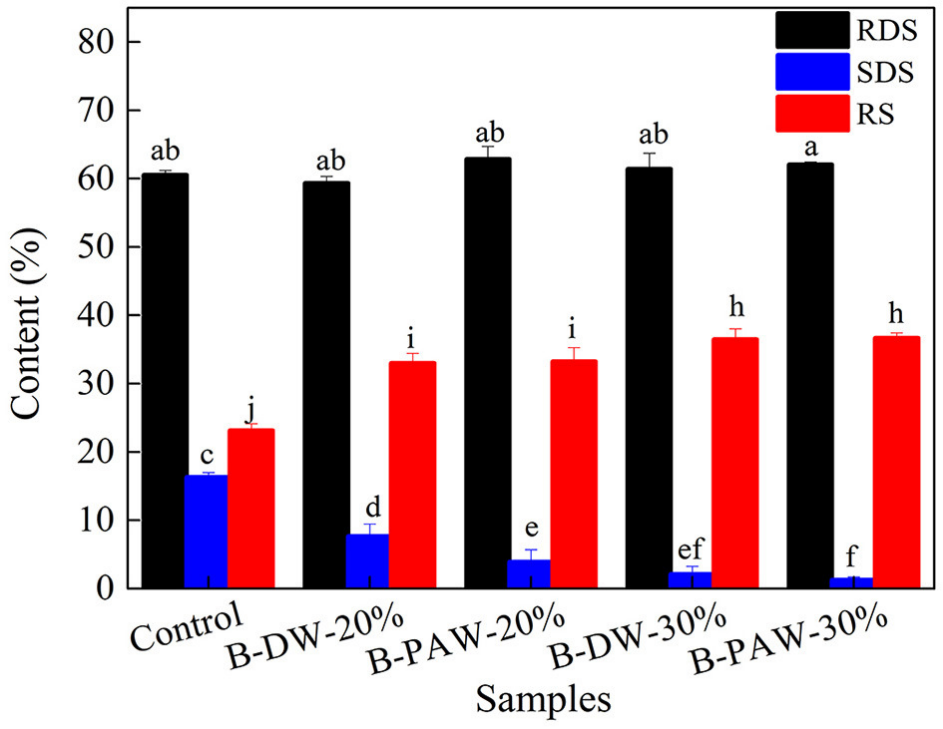
Effect of modified flour on the digestion of steamed bread. Different lowercase letters indicate significant differences at *p* < 0.05.

Slowly digestible starch (SDS) is related to slower glucose entering the blood and lower blood sugar response. SDS has a lower rate of complete digestion in the small intestine than rapidly digestible starch (RDS). However, resistant starch (RS) cannot be digested in the small intestine (27, 28). Studies have shown that intake of foods rich in RS was beneficial for preventing obesity, controlling blood sugar release and colon health (29). The effect of modified flour on the *in vitro* digestion of steamed bread is shown in Figure 4. It can be seen from the results that RS content of steamed bread in the control group was 23.16%, and SDS content was 16.31%. After the addition of modified flour, the RS content of steamed bread was significantly increased, while the SDS content was decreased. The RS content of steamed bread with 30% of PAW-15% was the highest, which reached 36.66%. It may be due to the formation of starch-lipid (protein) complexes during the HMT, which caused SDS to convert to RS. On the other hand, proteins were prone to denaturation when exposed to heat and acids. The denatured proteins may be distributed in starch particles and attached to their surfaces. This barrier can effectively reduce the contact between starch and amylase. According to the effect of modified flour on the in basic indicators of steamed bread, it can be seen that the volume of steamed bread with modified flour was reduced, and the internal structure was dense. The chance of contact between starch and enzyme decreased, which improved the anti-digestibility of steamed bread. Amylose has a helical structure and is easy to form amylose-lipid complex (30), which belongs to the fifth type of resistant starch (31). This may also be the reason for the increase of RS content in steamed bread with modified flour.

## Conclusion

The physicochemical properties of wheat flour were affected by HMT and PAW. Under the effect of high temperature and PAW, the crystal structure of wheat flour samples was damaged. With the increase of PAW treated wheat flour, the specific volume of steamed bread decreased and the spread ratio increased. The addition of PAW-HMT increased the hardness and chewiness of the steamed bread. On the other hand, the resistant starch content of steamed bread with the addition of PAW-HMT modified flour was significantly increased. However, excessive addition of HMT flour was not conducive to the quality of steamed bread, and excessive addition will reduce the acceptance. Therefore, it was recommended to add 20% PAW-15% flour to the steamed bread.

## Data Availability Statement

The original contributions presented in the study are included in the article/supplementary material, further inquiries can be directed to the corresponding author/s.

## Author Contributions

MS contributed to conception and design the study. YC and FW performed the experiments and analyzed the data. YC wrote the first draft of the manuscript. XJ, YL, and YY contributed to writing-review and editing and funding. All authors contributed to the article and approved the submitted version.

## Funding

We gratefully acknowledge financial support by Science and Technology Innovation Talents in Universities of Henan Province (20HASTIT037), Science and Technology Basic Research Program of Henan Province (202102110302, 222102110337).

## Conflict of Interest

FW was employed by Lanzhou Quality Supervision Center Limited, China Grain Reserves Group Ltd. Company. The remaining authors declare that the research was conducted in the absence of any commercial or financial relationships that could be construed as a potential conflict of interest.

## Publisher's Note

All claims expressed in this article are solely those of the authors and do not necessarily represent those of their affiliated organizations, or those of the publisher, the editors and the reviewers. Any product that may be evaluated in this article, or claim that may be made by its manufacturer, is not guaranteed or endorsed by the publisher.
